# Synthesis, Characterization, and *In Vitro* Anticancer Evaluation of Novel 2,5-Disubstituted 1,3,4-Oxadiazole Analogue

**DOI:** 10.1155/2014/491492

**Published:** 2014-08-10

**Authors:** Salahuddin, Avijit Mazumder, Mohammad Shaharyar

**Affiliations:** ^1^Department of Pharmaceutical Technology, Noida Institute of Engineering and Technology, Greater Noida, Uttar Pradesh 201306, India; ^2^Department of Pharmaceutical Chemistry, Faculty of Pharmacy, Jamia Hamdard (Hamdard University), New Delhi 110062, India

## Abstract

In this series, we have synthesised a new 2,5-disubstituted 1,3,4-oxadiazole in search of potential therapeutics for cancer. The anticancer activities were evaluated on a panel of 60 cell lines by the National Cancer Institute according to its own screening protocol. Out of the 24 compounds, 11 were selected and evaluated via single high dose (10^−5^ M). In the next phase, two compounds have been selected for five-dose assay. The compounds 3-(5-benzyl-1,3,4-oxadiazol-2-yl)quinolin-2(1H)-one **18** (NSC-776965) and 3-[5-(2-phenoxymethyl-benzoimidazol-1-ylmethyl)-[1,3,4]oxadiazol-2-yl]-2-p-tolyloxy-quinoline **27** (NSC-776971) showed mean growth percentage of 66.23 and 46.61, respectively, in one-dose assay and their GI_50_ values ranging between 1.41–15.8 *μ*M and 0.40–14.9 *μ*M, respectively, in 5-dose assay.

## 1. Introduction

**Scheme 1:**
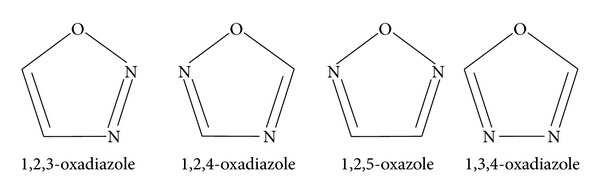


Cancer is primarily an environmental disease with 90–95% cases being related to environmental factors and 5–10% to genetics [1]. Common environmental factors causing cancer are tobacco (25–30%), diet and obesity (30–35%), infections (15–20%), radiation (both ionizing and nonionizing, up to 10%), stress, lack of physical activity, and environmental pollutant [2]. Death rates for cancer have continued to decline for both men and women of all racial and ethnic groups and have decreased by 1.5% per year from 2000–2009 for both sexes [3]. Trends in cancer death rates continue to decline; however, increase in incidence rates for some HPV associated cancer and low vaccination coverage among adolescents emphasize need for prevention in HPV associated cancer as well as to increase the coverage of vaccine. The development of new anticancer therapeutic agents is one of the fundamental goals in medicinal chemistry [4]. Medicinal chemists have great interest in research and development for the search of newer and safer anticancer agents. Epidermal growth factor receptor (EGFR) family of tyrosine kinase (TK) play a vital role in cancer proliferation and it is suggested that any agent would inhibit the TK activity and may have a considerable role in cancer treatment. N-containing heterocyclic specially 1,3,4-oxadiazole ring are of great interest for researchers as they are found in natural products and are used frequently in medicinal and pharmaceutical chemistry [5]. Oxadiazole has a furan ring with two methane (–CH=) groups and is replaced by two pyridine types of nitrogen (–N=) atoms. Four types of isomers are possible in oxadiazole nucleus depending on the position of nitrogen present in the ring [6]. (See Scheme 1).

The heterocyclic compounds containing 1,3,4-oxadiazole moiety are used as piconjugation which is used to prepare a large number of biologically active molecules (donor-acceptor) that carry a pielectron in their aromatic ring. Therefore, the compounds containing 1,3,4-oxadiazole moiety may be a good choice for optical material or biologically active chemicals [7]. A large number of therapeutic agents like HIV-integrase inhibitor raltegravir [8], nitrofuran antibacterial furamizole [9], antihypertensive agents tiodazosin [10], and nesapidil [11] are based on 1,3,4-oxadiazole moiety. For the development of pharmaceutically active compound, researcher have explored various molecules containing 1,3,4-oxadiazole nucleus which have already been explored. The literature review reveals that the compounds having five-membered heterocyclic ring containing nitrogen and oxygen like 1,3,4-oxadiazole have been synthesized and have showed a variety of biological activities like anticancer [12–16], anticonvulsant [17–19], antimicrobial [20–25], anti-inflammatory analgesic [26–29], dyes and pigments [30], ulcerogenic [31], antitubercular activities [32]. Apart from that there are some natural analogues like curcumin and its derivative which have very good anticancer activity [33].

## 2. Material and Method

### 2.1. Chemistry

The chemicals used for experimental work were commercially procured from various chemical units, namely, E. Merck India Ltd., CDH and S.D. Fine chem. and Qualigens. These solvent and reagents were of LR grade and were purified before use. The silica gel G (160-120 mesh) used for analytical chromatography (TLC) was obtained from E. Merck India Ltd. The solvent system used was benzene : acetone (9 : 1) and (8 : 2) and toluene : ethyl acetate : formic acid (5 : 4 : 1). Ashless Whattman number 1 filter paper was used for vacuum filtration. Melting points were determined in open glass capillary using melting point apparatus and are uncorrected. The proton nuclear magnetic resonance (^1^HNMR) spectra were recorded on Bruker 300 MHz instrument in DMSO-d_6_/CDCl_3_ using tetramethylsilane [(CH_3_)_4_Si] as internal standard. The infrared spectra of the compound were recorded in KBr on Perkin-Elmer FTIR Spectrometer, and the iodine chamber and U.V.-lamp were used for visualisation of TLC spots. Mass spectra were recorded on API 2000 LC/MS/MS system and elemental analyses were performed on Perkin-Elmer 2400. The commercially available grades of solvents and reagents were found to be of adequate purity. However, the presence of undesirable impurities and others was likely to be used for experimental work for purification.

### 2.2. Synthesis of 2-Chloroquinoline-3-carbaldehyde **(2)**

2-Chloroquinoline-3-carbaldehyde was synthesized from acetanilide* via* a Vilsmeier-Haack reaction or by traditional methods. To a solution of acetanilide (5 mmole) in dry DMF (15 mmole) at 0–5°C with stirring, phosphorous oxychloride (60 mmole) was added dropwise and the mixture was stirred at 80–90°C for time ranges between 4 and 15 hr. The mixture was poured into crushed ice and stirred well and the resulting solid substance was filtered, washed well with cold water, and dried. The compounds were purified by recrystallization from either ethyl acetate or acetonitrile. Yield 65%, yellow solid, m.p 146–148°C. IR (KBr) cm^−1^: 2871 (CH–ArH), 1687 (C=O), 749 (C–Cl). ^1^H-NMR (DMSO-d_6_) *δ* ppm: 6.78 (t, 1H, *J* = 7.1 Hz, H-5), 6.89 (d, 1H, *J* = 6.3 Hz, H-8), 7.25 (d, 1H, *J* = 6.5 Hz, H-6), 7.70 (t, 1H, *J* = 6.2, H-7), 10.25 (s, 1H, CHO). MS *m*/*z*: 191.01 (M^+^). Anal. Calc. for C_10_H_6_ClNO; C, 62.68; H, 3.16; Cl, 18.50; N, 7.31; O, 8.35; Found: C, 62.71; H, 3.18; Cl, 18.48; N, 7.33; O, 8.37. MS *m*/*z* (M^+^) 191.

#### 2.2.1. Synthesis of 2-Oxo-1,2-dihydroquinoline-3-carbaldehyde **(3)**

A suspension of aldehyde (5 mmol) in 70% acetic acid (50 mL) was heated under reflux for 4-5 h. The process of the reaction was checked by thin layer chromatography. Upon cooling the reaction mixture, a solid product precipitated, which was filtered, washed with water, and dried. Yield 90%. m.p. 300–304°C. IR (KBr) cm^−1^: 3320 (NH), 1670 (C=O), 2923 (CH aromatic). ^1^H-NMR (300 MHz DMSO-d_6_) *δ* ppm: 7.21 (t, 1H, *J* = 7.4 Hz, H-7), 7.37 (d, 1H, *J* = 6.3 Hz, H-8), 7.61 (t, 1H, *J* = 6.6 Hz, H-6), 7.80 (d, 1H, *J* = 6 Hz, H-5), 8.23 (s, 1H, H-4), 10.25 (CHO), 12.23 (CONH); Anal calcd for C_10_H_7_NO_2_: C 69.36, H 4.07, N 8.09 Found: C 69.13, H 3.99, N 7.93; MS *m*/*z* (M^+^) 173.

#### 2.2.2. Synthesis 2-(p-Tolyloxy)quinoline-3-carbaldehyde **(4)**

To a mixture of p-cresol (0.031 mmol) K_2_CO_3_ (0.068 mmol) in DMF, the 2-chloroquinoline-3-carbaldehyde (0.031 mmol) was added and the reaction mixture was stirred at 85–90°C for 5 h. The completion of reaction was monitored by TLC. After completion, water (50 mL) was poured in the reaction mixture and the solid thus obtained was filtered off and recrystallized from ethyl alcohol. Yield 76%; m.p. 126–128°C. IR (KBr) cm^−1^: 2950 (CH, aromatic), 2670 (CH, aliphatic), 1720 (C=O); 1230 (C–O). ^1^H-NMR (300 MHz DMSO-d_6_) *δ* ppm: 2.35 (s, 3H, CH_3_), 7.05 (d, 2H, *J* = 6.5 Hz, ArH), 7.19 (d, 2H, *J* = 6.6 Hz, ArH), 7.31–7.69 (m, 3H, ArH), 7.77 (d, 2H, *J* = 7.8 Hz, ArH), 10.35 (s, 1H, CHO). Anal calcd for C_17_H_13_NO_2_: C 77.55, H 4.98, N 5.32, O 12.15. Found C 77.58, H, 5.02, N 5.28, O 12.10; MS *m*/*z* (M^+^) 263.

### 2.3. General Method for the Synthesis of 2-(2-Phenoxy/naphthyloxy-1H-benzimidazol-1-yl)-N′-[(E)-(2-oxo-1,2-dihydroquinolin-3-yl)methylidene]acetohydrazide **(5-6)**

A mixture of 2-[2-(phenoxy/naphthalen-2-yloxy methyl)-1H-benzimidazol-1-yl]acetohydrazide [12, 19] and 2-oxo-1,2-dihydroquinoline-3-carbaldehyde** (3)** in ethanol was refluxed for 5 h. After completion of the reaction, the reaction mixture was concentrated, cooled, and poured in ice cold water, and the precipitate so formed was filtered, dried, and recrystallized to give the desired compound.

#### 2.3.1. (2-Phenoxy Methyl-benzoimidazol-1-yl)-acetic acid (2-oxo-1,2-dihydro-quinolin-3-ylmethylene)-hydrazide **(5)**

Yield 73%, m.p. 296–299°C. IR (KBr) cm^−1^: 3299 (NH), 2856 (CH, aromatic), 1647 (C=O), 1455 (N=CH), 1260 (C–O). ^1^H-NMR (300 MHz DMSO-d_6_) *δ* ppm: 4.91 (s, 2H, CH_2_), 5.31 (s, 2H, CH_2_O), 7.17–7.24 (m, 3H, ArH), 7.33 (t, 3H, *J* = 15.6 Hz, ArH), 7.46 (t, 2H, *J* = 12.9 Hz, ArH), 7.62 (t, 2H, *J* = 15 Hz, ArH), 7.71–7.91 (m, 4H, ArH), 8.43 (N=CH), 10.24 (s, 1H, CH_2_CONH), 12.12 (s, 1H, CONH quinoline). Anal calcd for C_26_H_21_N_5_O_3_: C 69.17, H 4.69, N 15.51, O 10.63. Found: C 69.11, H 4.73, N 15.54, O 10.59; MS *m*/*z* (M^+^) 451.

#### 2.3.2. [2-(Naphthalen-2-yloxymethyl)-benzoimidazol-1-yl]-acetic acid (2-oxo-1,2-dihydro-quinolin-3-ylmethylene)-hydrazide **(6)**

Yield 73%, m.p. 216–219°C. IR (KBr) cm^−1^: 3170 (NH), 2871 (CH, aromatic), 1678 (C=O), 1447 (N=CH). ^1^H-NMR (300 MHz DMSO-d_6_) *δ* ppm: 4.95 (s, 2H, CH_2_), 5.29 (s, 2H, CH_2_O naphthyloxy), 7.12–7.21 (m, 4H, ArH), 7.32 (t, 3H, *J* = 15.3 Hz, ArH), 7.46 (t, 2H, *J* = 12.3 Hz, ArH), 7.59 (t, 2H, *J* = 14.4 Hz, ArH), 7.63–7.87 (m, 5H, ArH), 8.33 (N=CH), 10.21 (s, 1H, CH_2_CONH), 12.22 (s, 1H, CONH quinoline). Anal calcd for C_30_H_23_N_5_O_3_: C 71.84, H 4.62, N 13.96, O 9.57. Found: C 71.87, H 4.59, N 13.95, O 9.57; MS *m*/*z* (M^+^) 501.

### 2.4. General Method for the Synthesis of 3-[5-(2-Phenoxymethyl/naphthyloxy-benzoimidazol-1-ylmethyl)-[1,3,4]oxadiazol-2-yl]-1H-quinolin-2-one **(7, 8)**

To an ethanolic solution of 2-(2-phenoxy/naphthyloxy-1H-benzimidazol-1-yl)-N′-[(E)-(2-oxo-1,2-dihydroquinolin-3-yl)methylidene]acetohydrazide (0.01 mole)** (5-6)** and chloramin-T (0.01 mole) was added. The solution was refluxed for 4 h, and sodium chloride which separated out during the course of reaction was filtered off. Excess ethanol was completely removed from the filtrate by distillation under reduced pressure, leaving behind a solid mass which was crystallized from ethanol to give the desired compound. Recrystallize the compounds from ethyl alcohol.

#### 2.4.1. 3-[5-(2-Phenoxymethyl-benzoimidazol-1-ylmethyl)-[1,3,4]oxadiazol-2-yl]-1H-quinolin-2-one **(7)**

Yield 58%. m.p. 252–255°C. IR (KBr) cm^−1^: 3169 (NH), 2891 (CH, aromatic), 1667 (C=O), 1243 (C–O). ^1^H-NMR (300 MHz DMSO-d_6_) *δ* ppm: 5.25 (s, 2H, CH_2_), 5.51 (s, 2H, CH_2_O), 6.91 (t, 1H, *J* = 14.1 Hz, ArH), 7.01 (d, 2H, *J* = 7.5 Hz ArH), 7.21–7.37 (m, 8H, ArH), 7.60–7.71 (m, 4H, ArH), 7.81 (d, 1H, *J* = 7.5 Hz, ArH), 12.47 (s, 1H, CONH). Anal calcd for C_26_H_19_N_5_O_3_: C 69.48, H 4.26, N 15.58, O 10.68. Found: C 69.51, H 4.21, N 15.52, O 10.69; MS *m*/*z* (M^+^) 449.

#### 2.4.2. 3-[5-(2-Naphthyloxymethyl-benzoimidazol-1-ylmethyl)-[1,3,4]oxadiazol-2-yl]-1H-quinolin-2-one **(8)**

Yield 61%. m.p. 222–225°C. IR (KBr) cm^−1^: 3172 (NH), 2890 (CH, aromatic), 1662 (C=O), 1244 (C–O). ^1^H-NMR (300 MHz DMSO-d_6_) *δ* ppm: 5.22 (s, 2H, CH_2_), 5.61 (s, 2H, CH_2_O), 6.87 (t, 2H, *J* = 14.1 Hz, ArH), 7.03 (d, 2H, *J* = 7.5 Hz ArH), 7.20–7.36 (m, 9H, ArH), 7.49–7.78 (m, 4H, ArH), 7.84 (d, 1H, *J* = 7.7 Hz, ArH), 12.42 (s, 1H, CONH). Anal calcd for C_30_H_21_N_5_O_3_: C 72.13, H 4.24, N 14.02, O 9.61. Found: C 72.08, H 4.28, N 13.97, O 9.65; MS *m*/*z* (M^+^) 499.

### 2.5. General Method for the Synthesis of 2-(Substituted)-(2-oxo-1,2-dihydroquinolin-3-yl)methylidene]acetohydrazide **(9–16)**

A mixture of aromatic hydrazide [34] and 2-oxo-1,2-dihydroquinoline-3-carbaldehyde** (3)** in ethanol was refluxed for 5 h. After completion of the reaction, the reaction mixture was concentrated, cooled, and poured in ice cold water, and the precipitate so formed was filtered, dried, and recrystallized to give the desired compound.

#### 2.5.1. 2-(Naphthalen-2-yloxy)-N′-[(E)-(2-oxo-1,2-dihydroquinolin-3-yl)methylidene]acetohydrazide **(9)**

Yield 64%. m.p. 236–239°C. IR (KBr) cm^−1^: 3304 (NH), 2921 (CH, aromatic), 1666 (C=O), 1277 (C–O). ^1^H-NMR (300 MHz DMSO-d_6_) *δ* ppm: 5.33 (s, 2H, CH_2_O), 7.15–7.33 (m, 4H, ArH), 7.45 (d, 2H, *J* = 10.5 Hz, ArH), 7.83 (t, 2H, *J* = 17.8 Hz, ArH), 7.53–7.71 (m, 3H, ArH), 8.01 (s, 1H, ArH), 8.43 (s, 1H, N=CH), 10.54 (s, 1H, CH_2_CONH), 11.89 (s, 1H, CONH quinoline). Anal calcd for C_23_H_19_N_3_O_2_: C 71.15, H 4.61, N 11.31, O 12.92. Found C 71.11, H 4.66, N 11.26, O 12.95; MS *m*/*z* (M^+^) 385.

#### 2.5.2. N′-[(E)-(2-Oxo-1,2-dihydroquinolin-3-yl)methylidene]-2-phenylacetohydrazide **(10)**

Yield 53%. m.p. 212–216°C. IR (KBr) cm^−1^: 3274 (NH), 2923 (CH, aromatic), 1666 (C=O), 1500 (N=C), 1305 (C–O). ^1^H-NMR (300 MHz DMSO-d_6_) *δ* ppm: 5.64 (s, 2H, OCH_2_), 6.89 (s, 1H, ArH), 7.06 (d, 2H, *J* = 6 Hz, ArH), 7.33 (t, 4H, *J* = 8.1 Hz, ArH), 7.50–7.68 (m, 3H, ArH), 7.71–7.81 (m, 1H, ArH), 8.29 (s, 1H, N=CH), 8.48 (s, 1H, CH_2_CONH), 11.81 (s, 1H, CONH quinoline). Anal calcd for C_18_H_15_N_3_O_2_: C 70.81, H 4.95, N 13.76, O 10.48. Found C 70.85, H 4.89, N 13.79, O 10.53; MS *m*/*z* (M^+^) 319.

#### 2.5.3. 4-Nitro-N′-[(E)-(2-oxo-1,2-dihydroquinolin-3-yl)methylidene]benzohydrazide **(11)**

Yield 57%. m.p. 294–298°C. IR (KBr) cm^−1^: 3214 (NH), 2931 (CH, aromatic), 1670 (C=O), 1503 (N=C), 1303 (C–O). ^1^H-NMR (300 MHz DMSO-d_6_) *δ* ppm: 7.05 (d, 2H, *J* = 6.9 Hz, ArH), 7.20–7.33 (m, 5H, ArH), 7.41 (t, 1H, *J* = 22.6 Hz, ArH), 7.78 (d, 2H, *J* = 7.5 Hz ArH), 8.37 (s, 1H, N=CH), 11.45 (s, 1H, CONH), 11.93 (s, 1H, CONH quinoline). Anal calcd for C_17_H_12_N_4_O_4_: C 60.71, H 3.60, N 16.66, O 19.03. Found: C 60.65, H 3.66, N 16.70, O 19.02; MS *m*/*z* (M^+^) 350.

#### 2.5.4. 4-Chloro-N′-[(E)-(2-oxo-1,2-dihydroquinolin-3-yl)methylidene]benzohydrazide **(12)**

Yield 57%. m.p. 284–286°C. IR (KBr) cm^−1^: 3266 (NH), 2922 (CH, aromatic), 1670 (C=O), 1491 (N=C), 1311 (C–O). ^1^H-NMR (300 MHz DMSO-d_6_) *δ* ppm: 7.19–7.23 (m, 2H, ArH), 7.32–7.35 (m, 2H, ArH), 7.51 (t, 2H, *J* = 15 Hz, ArH), 7.59 (d, 1H, *J* = 8.1 Hz, ArH), 7.84 (d, 2H, *J* = 7.2 Hz, ArH), 7.95 (d, 2H, *J* = 8.4 Hz, ArH), 8.47 (s, 1H, N=CH), 8.71 (s, 1H, CONH), 12.02 (s, 1H, CONH quinoline). Anal calcd for C_17_H_12_ClN_3_O_2_: C 62.68, H 3.71, Cl 10.88, N 12.90, O 9.82. Found: C 62.72, H 3.75, Cl 10.91, N 12.93, O 9.85; MS *m*/*z* (M^+^) 339.

#### 2.5.5. N′-[(E)-(2-Oxo-1,2-dihydroquinolin-3-yl)methylidene]pyridine-3-carbohydrazide **(13)**

Yield 57%. m.p. 268-269°C. IR (KBr) cm^−1^: 3305 (NH), 2922 (CH, aromatic), 1604 (C=O), 1513 (N=C), 1277 (C–O). ^1^H-NMR (300 MHz DMSO-d_6_) *δ* ppm: 7.14–7.50 (m, 6H, ArH), 7.70 (d, 2H, *J* = 7.8 Hz, ArH), 7.92 (t, 1H, *J* = 24 Hz, ArH), 8.66 (s, 1H, N=CH), 10.24 (s, 1H, CONH), 11.71 (s, 1H, CONH quinoline). Anal calcd for C_16_H_12_N_4_O_2_: C 65.75, H 4.14, N 19.17, O 10.95. Found: C 65.70, H 4.17, N 19.17, O 10.99; MS *m*/*z* (M^+^) 306.

#### 2.5.6. N′-[(E)-(2-Oxo-1,2-dihydroquinolin-3-yl)methylidene]-2-phenoxyacetohydrazide **(14)**

Yield 67%. m.p. 85–88°C. IR (KBr) cm^−1^: 3255 (NH), 2945 (CH, aromatic), 1634 (C=O), 1500 (N=C), 1306 (C–O). ^1^H-NMR (300 MHz DMSO-d_6_) *δ* ppm: 4.46 (s, 2H, OCH_2_), 7.31–7.37 (t, 4H, *J* = 15.6 Hz, ArH), 7.38 (s, 1H, aromatic), 7.48–7.61 (m, 5H, ArH), 8.30 (s, 1H, N=CH), 10.31 (s, 1H, CONH), 11.29 (s, 1H, CONH quinoline). Anal calcd for C_16_H_12_N_4_O_2_: C 67.28, H 4.71, N 13.08, O 14.94. Found: C 67.32, H 4.69, N 13.02, O 14.97; MS *m*/*z* (M^+^) 306.

#### 2.5.7. 3,5-Dinitro-N′-[(E)-(2-oxo-1,2-dihydroquinolin-3-yl)methylidene]benzohydrazide **(15)**

Yield 72%. m.p. 270–272°C. IR (KBr) cm^−1^: 3311 (NH), 2931 (CH, aromatic), 1663 (C=O), 1500 (N=C), 1309 (C–O). ^1^H-NMR (300 MHz DMSO-d_6_) *δ* ppm: 6.96 (t, 2H, *J* = 21.3 Hz, ArH), 7.14–7.34 (m, 3H. ArH), 7.50 (d, 1H, *J* = 7.5 Hz, ArH), 7.75 (d, 1H, *J* = 7.5 Hz, ArH), 8.20 (s, 1H, ArH), 8.58 (s, 1H, N=CH), 11.67 (s, 1H, CONH), 11.98 (s, 1H, CONH quinoline). Anal calcd for C_17_H_11_N_5_O_6_: C 53.55, H 2.91, N 18.37, O 25.18. Found: C 53.50, H 2.94, N 18.39, O 25.18; MS *m*/*z* (M^+^) 380.

#### 2.5.8. 2-Hydroxy-N′-[(E)-(2-oxo-1,2-dihydroquinolin-3-yl)methylidene]benzohydrazide **(16)**

Yield 71%. m.p. 149–151°C. IR (KBr) cm^−1^: 3307 (NH), 2930 (CH, aromatic), 1634 (C=O), 1500 (N=C), 1308 (C–O). ^1^H-NMR (300 MHz DMSO-d_6_) *δ* ppm: 6.88 (m, 2H, ArH), 7.23 (t, 1H, *J* = 15 Hz, ArH), 7.33 (d, 1H, *J* = 8.4 Hz. ArH), 7.46 (t, 2H, *J* = 15.3 Hz, ArH), 7.61 (t, 1H, *J* = 15.6 Hz, ArH), 7.76 (d, 1H, *J* = 7.8 Hz, ArH), 7.89 (d, 1H, *J* = 7.8 Hz, ArH), 8.49 (s, 1H, N=CH), 10.22 (s, 1H, OH), 11.29 (s, 1H, CONH), 12.22 (s, 1H, CONH quinoline). Anal calcd for C_17_H_13_N_3_O_3_: C 66.44, H 4.26, N 13.67, O 15.62. Found: C 66.47, H 4.31, N 13.74, O 15.65; MS *m*/*z* (M^+^) 321.

### 2.6. General Method for the Synthesis of 3-(5-Substituted-1,3,4-oxadiazol-2-yl)quinolin-2(1H)-one **(17–24)**

To an ethanolic solution of 2-(substituted)-(2-oxo-1,2-dihydroquinolin-3-yl)methylideneacetohydrazide (0.01 mole)** (9-16)** and chloramin-T (0.01 mole) was added. The solution was refluxed for 4 h, and sodium chloride which separated out during the course of reaction was filtered off. Excess ethanol was completely removed from the filtrate by distillation under reduced pressure, leaving behind a solid mass which was crystallized from ethanol to give the desired compound. Recrystallize from ethyl alcohol.

#### 2.6.1. 3-{5-[(Naphthalen-2-yloxy)methyl]-1,3,4-oxadiazol-2-yl}quinolin-2(1H)-one **(17)**

Yield 61%. m.p. 116–124°C. IR (KBr) cm^−1^: 3317 (NH), 2934 (CH, aromatic), 1636 (C=O), 1441 (N=C), 1333 (C–O). ^1^H-NMR (300 MHz DMSO-d_6_) *δ* ppm: 5.22 (s, 2H, OCH_2_), 7.34 (D, 2H, *J* = 8.1 Hz, ArH), 7.40–7.63 (m, 8H, ArH), 7.68 (d, 1H, *J* = 8.1 Hz, ArH), 11.23 (s, 1H, CONH). Anal calcd for C_22_H_15_N_3_O_2_: C 74.78, H 4.28, N 11.89, O 9.06. Found: C 74.77, H 4.31, N 11.84, O 9.06; MS *m*/*z* (M^+^) 369.

#### 2.6.2. 3-(5-Benzyl-1,3,4-oxadiazol-2-yl)quinolin-2(1H)-one **(18)**

Yield 64%. m.p. 189-190°C. IR (KBr) cm^−1^: 3382 (NH), 2904 (CH, aromatic), 1656 (C=O), 1435 (N=C), 1305 (C–O). ^1^H-NMR (300 MHz DMSO-d_6_) *δ* ppm: 4.36 (s, 2H, CH_2_), 7.27 (t, 5H, *J* = 15 Hz, ArH), 7.46–7.70 (m, 3H, ArH), 7.79–7.91 (m, 2H, ArH), 12.17 (s, 1H, CONH). Anal calcd for C_18_H_13_N_3_O_2_: C 71.28, H 4.32, N 13.85, O 10.55. Found: C 71.23, H 4.37, N 13.85, O 10.59; MS *m*/*z* (M^+^) 303.

#### 2.6.3. 3-[5-(4-Nitrophenyl)-1,3,4-oxadiazol-2-yl]quinolin-2(1H)-one **(19)**

Yield 54%. m.p. 223–225°C. IR (KBr) cm^−1^: 3304 (NH), 2932 (CH, aromatic), 1665 (C=O), 1502 (N=C), 1304 (C–O). ^1^H-NMR (300 MHz DMSO-d_6_) *δ* ppm: 6.46 (t, 1H, *J* = 12.9 Hz, ArH), 7.00–7.38 (m, 3H, ArH), 7.50–7.93 (m, 5H, ArH), 12.17 (s, 1H, CONH). Anal calcd for C_17_H_10_N_4_O_4_: C 61.08, H 3.02, N 16.06, O 19.14. Found: C 61.13, H 3.08, N 16.04, O 19.11; MS *m*/*z* (M^+^) 348.

#### 2.6.4. 3-[5-(4-Chlorophenyl)-1,3,4-oxadiazol-2-yl]quinolin-2(1H)-one **(20)**

Yield 54%. m.p. 256–260°C. IR (KBr) cm^−1^: 3300 (NH), 2903 (CH, aromatic), 1650 (C=O), 1505 (N=C), 1310 (C–O). ^1^H-NMR (300 MHz DMSO-d_6_) *δ* ppm: 7.62–7.73 (m, 6H, ArH), 7.91 (t, 2H, *J* = 15.9 Hz, ArH), 8.09 (d, 1H, *J* = 7.8 Hz, ArH), 12.27 (s, 1H, CONH). Anal calcd for C_17_H_10_ClN_3_O_2_: C 63.07, H 3.11, Cl 10.95, N 12.98, O 9.88. Found: C 63.11, H 3.05, Cl 10.99, N 13.04, O 9.85; MS *m*/*z* (M^+^) 337.

#### 2.6.5. 3-[5-(Pyridin-3-yl)-1,3,4-oxadiazol-2-yl]quinolin-2(1H)-one **(21)**

Yield 67%. m.p. 168-169°C. IR (KBr) cm^−1^: 3159 (NH), 2971 (CH, aromatic), 1658 (C=O), 1491 (N=C), 1331 (C–O). ^1^H-NMR (300 MHz DMSO-d_6_) *δ* ppm: 7.17 (t, 2H, *J* = 14.1 Hz, ArH), 7.27 (d, 2H, *J* = 7.2 Hz, ArH), 7.48 (t, 2H, *J* = 14.4 Hz, ArH), 7.72 (d, 2H, *J* = 7.5 Hz, ArH), 7.93 (s, 1H, ArH), 11.82 (s, 1H, CONH). Anal calcd for C_16_H_10_N_4_O_2_: C 66.20, H 3.47, N 19.30, O 11.02. Found: C 66.23, H 3.41, N 19.33, O 11.06; MS *m*/*z* (M^+^) 304.

#### 2.6.6. 3-[5-(Phenoxymethyl)-1,3,4-oxadiazol-2-yl]quinolin-2(1H)-one **(22)**

Yield 67%. m.p. 137–140°C. IR (KBr) cm^−1^: 3090 (NH), 2915 (CH, aromatic), 1655 (C=O), 1429 (N=C), 1303 (C–O). ^1^H-NMR (300 MHz DMSO-d_6_) *δ* ppm: 5.23 (s, 2H, CH_2_O), 7.07 (t, 2H, *J* = 14.4 Hz, ArH), 7.27–7.46 (m, 5H, ArH), 7.74 (d, 2H, *J* = 7.5 Hz, ArH), 7.89 (s, 1H, ArH), 11.87 (s, 1H, CONH). Anal calcd for C_18_H_13_N_3_O_3_: C 67.71, H 4.10, N 13.16, O 15.03. Found: C 67.75, H 4.13, N 13.21, O 15.01; MS *m*/*z* (M^+^) 319.

#### 2.6.7. 3-[5-(3,5-Dinitrophenyl)-1,3,4-oxadiazol-2-yl]quinolin-2(1H)-one **(23)**

Yield 67%. m.p. 275–277°C. IR (KBr) cm^−1^: 3290 (NH), 2916 (CH, aromatic), 1665 (C=O), 1432 (N=C), 1305 (C–O). ^1^H-NMR (300 MHz DMSO-d_6_) *δ* ppm: 7.27–7.39 (m, 3H, ArH), 7.54 (t, 2H, *J* = 13.8 Hz, ArH), 7.81 (d, 2H, *J* = 6.9 Hz, ArH), 8.01 (s, 1H, ArH), 11.58 (s, 1H, CONH). Anal calcd for C_17_H_9_N_5_O_6_: C 53.86, H 2.39, N 18.46, O 25.31. Found: C 53.87, H 2.42, N 18.47, O 25.35; MS *m*/*z* (M^+^) 379.

#### 2.6.8. 3-[5-(2-Hydroxyphenyl)-1,3,4-oxadiazol-2-yl]quinolin-2(1H)-one **(24)**

Yield 74%. m.p. 288–290°C. IR (KBr) cm^−1^: 3307 (NH), 3167 (OH), 2936 (CH, aromatic), 1668 (C=O), 1432 (N=C), 1303 (C–O). ^1^H-NMR (300 MHz DMSO-d_6_) *δ* ppm: 6.88–6.94 (m, 3H, ArH), 7.13 (t, 2H, *J* = 15 Hz, ArH), 7.30 (d, 2H, *J* = 8.1 Hz, ArH), 7.43–7.51 (m, 4H, ArH), 7.60 (d, 1H, *J* = 8.1 Hz, ArH), 10.31 (s, 1H, OH), 12.23 (s, 1H, CONH). Anal calcd for C_17_H_11_N_3_O_3_: C 66.68, H 3.63, N 13.76, O 15.72. Found: C 66.61, H 3.68, N 13.79, O 15.65; MS *m*/*z* (M^+^) 305.

### 2.7. General Method for the Synthesis of 2-(2-Phenoxymethyl/naphthyloxymethyl-1H-benzimidazol-1-yl)-acetic acid (2-p-tolyloxy-quinolin-3-ylmethylene)-hydrazide **(25-26)**

A mixture of 2-[2-(phenoxy/naphthalen-2-yloxy methyl)-1H-benzimidazol-1-yl]acetohydrazide [12, 19] and 2-(p-tolyloxy)quioline-3-carbaldehyde** (4)** in ethanol was refluxed for 5 h. After completion of the reaction, the reaction mixture was concentrated, cooled, and poured in ice cold water, and the precipitate so formed was filtered, dried, and recrystallized to give the desired compound.

#### 2.7.1. (2-Phenoxy methyl-benzimidazol-1-yl)-acetic acid (2-p-tolyloxy-quinolin-3-ylmethylene)-hydrazide **(25)**

Yield 68%, m.p. 126–129°C. IR (KBr) cm^−1^: 3299 (NH), 2855 (CH, aromatic), 1665 (C=O), 1451 (N=CH), 1266 (C–O). ^1^H-NMR (300 MHz DMSO-d_6_) *δ* ppm: 2.37 (s, 3H, CH_3_), 5.47 (s, 2H, CH_2_), 5.62 (s, 2H, CH_2_O), 6.92 (t, 2H, *J* = 13.8 Hz, ArH), 7.03 (d, 3H, *J* = 7.2 Hz, ArH), 7.12–7.36 (m, 4H, ArH), 7.45 (t, 3H, *J* = 13.2 Hz, ArH), 7.54–7.63 (m, 2H, ArH), 7.73–7.92 (m, 4H, ArH), 8.28 (N=CH), 9.84 (s, 1H, CH_2_CONH). Anal calcd for C_33_H_27_N_5_O_3_: C 73.18, H 5.02, N 12.93, O 8.86. Found: C 73.12, H 5.04, N 12.96, O 8.89; MS *m*/*z* (M^+^) 591.

#### 2.7.2. (2-Naphthyloxymethyl-benzimidazol-1-yl)-acetic acid (2-p-tolyloxy-quinolin-3-ylmethylene)-hydrazide **(26)**

Yield 68%, m.p. 224–226°C. IR (KBr) cm^−1^: 3294 (NH), 2844 (CH, aromatic), 1684 (C=O), 1449 (N=CH), 1268 (C–O). ^1^H-NMR (300 MHz DMSO-d_6_) *δ* ppm: 2.34 (s, 3H, CH_3_), 5.34 (s, 2H, CH_2_), 5.77 (s, 2H, CH_2_O), 6.94 (t, 3H, *J* = 13.8 Hz, ArH), 7.08 (d, 3H, *J* = 7.4 Hz, ArH), 7.11–7.35 (m, 4H, ArH), 7.43 (t, 3H, *J* = 13.6 Hz, ArH), 7.47–7.60 (m, 3H, ArH), 7.67–7.86 (m, 4H, ArH), 8.34 (N=CH), 10.84 (s, 1H, CH_2_CONH). Anal calcd for C_37_H_29_N_5_O_3_: C 75.11, H 4.94, N 11.84, O 8.11. Found: C 75.15, H 4.97, N 11.80, O 8.12; MS *m*/*z* (M^+^) 541.

### 2.8. General Method for the Synthesis of 3-[5-(2-Phenoxymethyl/naphthyloxymethyl-benzoimidazol-1-ylmethyl)-[1,3,4]oxadiazol-2-yl]-2-p-tolyloxy-quinoline **(27-28)**

To an ethanolic solution of* 2-(2-phenoxymethyl/naphthyloxymethyl-1H-benzimidazol-1-yl)-acetic acid (2-p-tolyloxy-quinolin-3-ylmethylene)-hydrazide* (0.01 mole)** (25-26)** and chloramin-T (0.01 mole) was added. The solution was refluxed for 4 h, and sodium chloride which separated out during the course of reaction was filtered off. Excess ethanol was completely removed from the filtrate by distillation under reduced pressure, leaving behind a solid mass which was crystallized from ethanol to give the desired compound. Recrystallize from ethyl alcohol.

#### 2.8.1. 3-[5-(2-Phenoxymethyl-benzoimidazol-1-ylmethyl)-[1,3,4]oxadiazol-2-yl]-2-p-tolyloxy-quinoline **(27)**

Yield 68%. m.p. 80–84°C. IR (KBr) cm^−1^: 3202 (NH), 2799 (CH, aromatic), 1677 (C=O), 1405 (C=N), 1245 (C–O). ^1^H-NMR (300 MHz DMSO-d_6_) *δ* ppm: 2.34 (s, 3H, CH_3_), 5.48 (s, 2H, CH_2_), 6.07 (s, 2H, CH_2_O), 6.86–7.09 (m, 3H, ArH), 7.21 (d, 7H, *J* = 11.1 Hz ArH), 7.45–7.69 (m, 4H, ArH), 7.72 (t, 3H, *J* = 14.4 Hz, ArH), 8.14 (s, 1H, ArH). Anal calcd for C_33_H_25_N_5_O_3_: C 73.46, H 4.67, N 12.98, O 8.90. Found: C 73.49, H 4.62, N 12.94, O 8.96; MS *m*/*z* (M^+^) 539.

#### 2.8.2. 3-[5-(2-Naphthyloxy-methyl-benzoimidazol-1-ylmethyl)-[1,3,4]oxadiazol-2-yl]-2-p-tolyloxy-quinoline **(28)**

Yield 68%. m.p. 212–215°C. IR (KBr) cm^−1^: 3210 (NH), 2791 (CH, aromatic), 1674 (C=O), 1407 (C=N), 1249 (C–O). ^1^H-NMR (300 MHz DMSO-d_6_) *δ* ppm: 2.31 (s, 3H, CH_3_), 5.34 (s, 2H, CH_2_), 5.94 (s, 2H, CH_2_O), 6.82–7.11 (m, 4H, ArH), 7.18 (d, 7H, *J* = 11.4 Hz ArH), 7.38–7.71 (m, 5H, ArH), 7.77 (t, 3H, *J* = 15.3 Hz, ArH), 8.04 (s, 1H, ArH). Anal calcd for C_37_H_27_N_5_O_3_: C 75.37, H 4.62, N 11.88, O 8.14. Found: C 75.40, H 4.57, N 11.93, O 8.17; MS *m*/*z* (M^+^) 589.

### 2.9. General Method for the Synthesis of Substituted-(2-p-tolyloxy-quinolin-3-ylmethylene)-hydrazide **(29–36)**

A mixture of aromatic hydrazide [34] and 2-(p-tolyloxy)quioline-3-carbaldehyde** (4)** in ethanol was refluxed for 5 h. After completion of the reaction, the reaction mixture was concentrated, cooled, and poured in ice cold water, and the precipitate so formed was filtered, dried, and recrystallized to give the desired compound.

#### 2.9.1. (Naphthalen-2-yloxy)-acetic acid (2-p-tolyloxy-quinolin-3-ylmethylene)-hydrazide **(29)**

Yield 64%. m.p. 226-227°C. IR (KBr) cm^−1^: 3298 (NH), 2923 (CH, aromatic), 1667 (C=O), 1455 (C=N), 1277 (C–O). ^1^H-NMR (300 MHz DMSO-d_6_) *δ* ppm: 2.36 (s, 3H, CH_3_), 4.83 (s, 2H, OCH_2_), 7.15–7.37 (d, 4H, *J* = 8.4 Hz, ArH), 7.48 (d, 4H, *J* = 7.5 Hz, ArH), 7.57–7.68 (m, 4H, ArH), 7.82 (t, 4H, *J* = 18.3 Hz, ArH), 8.53 (s, 1H, N=CH), 11.82 (s, 1H, CH_2_CONH). Anal calcd for C_29_H_23_N_3_O_3_: C 75.47, H 5.02, N 9.10, O 10.40. Found C 75.49, H 5.08, N 9.14, O 10.45; MS *m*/*z* (M^+^) 461.

#### 2.9.2. Phenylacetic acid (2-p-tolyloxy-quinolin-3-ylmethylene)-hydrazide **(30)**

Yield 64%. m.p. 228-229°C. IR (KBr) cm^−1^: 3290 (NH), 2917 (CH, aromatic), 1666 (C=O), 1458 (C=N), 1260 (C–O). ^1^H-NMR (300 MHz DMSO-d_6_) *δ* ppm: 2.32 (s, 3H, CH_3_), 4.08 (s, 2H, CH_2_), 7.02 (d, 2H, *J* = 9 Hz, ArH), 7.14–7.32 (m, 5H, ArH), 7.51–7.60 (m, 2H, ArH), 7.46 (t, 1H, *J* = 18.3 Hz, ArH), 8.34 (s, 1H, N=CH), 10.61 (s, 1H, CH_2_CONH). Anal calcd for C_25_H_21_N_3_O_2_: C 75.93, H 5.35, N 10.63, O 8.09. Found C 75.99, H 5.37, N 10.63, O 8.03; MS *m*/*z* (M^+^) 395.

#### 2.9.3. N′-{(E)-[2-(4-Methylphenoxy)quinolin-3-yl]methylidene}-4-nitrobenzohydrazide **(31)**

Yield 64%. m.p. 151–153°C. IR (KBr) cm^−1^: 3293 (NH), 2927 (CH, aromatic), 1664 (C=O), 1457 (C=N), 1263 (C–O). ^1^H-NMR (300 MHz DMSO-d_6_) *δ* ppm: 3.79 (s, 3H, CH_3_), 7.17–7.29 (m, 4H, ArH), 7.50–7.64 (m, 3H, ArH), 8.10 (d, 2H, *J* = 7.8 Hz, ArH), 8.19 (d, 2H, ArH), 8.36 (d, 2H, *J* = 8.1 Hz, ArH), 8.91 (s, 1H, N=CH), 12.39 (s, 1H, CONH). Anal calcd for C_24_H_18_N_4_O_4_: C 67.60, H 4.25, N 13.14, O 15.01. Found C 67.63, H 4.29, N 13.19, O 15.00; MS *m*/*z* (M^+^) 426.

#### 2.9.4. 4-Chlorobenzoic acid (2-p-tolyloxy-quinolin-3-ylmethylene)-hydrazide **(32)**

Yield 67%. m.p. 244-245°C. IR (KBr) cm^−1^: 3198 (NH), 2908 (CH, aromatic), 1667 (C=O), 1460 (C=N), 1261 (C–O), 728 (Cl). ^1^H-NMR (300 MHz DMSO-d_6_) *δ* ppm: 2.36 (s, 3H, CH_3_), 7.16 (d, 2H, *J* = 8.1 Hz, ArH), 7.26 (d, 2H, *J* = 8.4 Hz, ArH), 7.49 (t, 2H, *J* = 14.7 Hz, ArH), 7.59–7.69 (m, 4H, ArH), 7.98 (d, 2H, *J* = 8.4 Hz, ArH), 8.07 (s, 1H, ArH), 8.72 (s, 1H, N=CH), 12.07 (s, 1H, CONH). Anal calcd for C_24_H_18_ClN_3_O_2_: C 69.31, H 4.36, Cl 10.10, N 10.10, O 7.69. Found C 69.35, H 4.41, Cl 10.13, N 10.05, O 7.75; MS *m*/*z* (M^+^) 415.

#### 2.9.5. N′-{(E)-[2-(4-Methylphenoxy)quinolin-3-yl]methylidene}pyridine-3-carbohydrazide **(33)**

Yield 71%. m.p. 88–90°C. IR (KBr) cm^−1^: 3298 (NH), 2913 (CH, aromatic), 1665 (C=O), 1467 (C=N), 1256 (C–O). ^1^H-NMR (300 MHz DMSO-d_6_) *δ* ppm: 3.27 (s, 3H, CH_3_), 7.10 (d, 2H, *J* = 8.1 Hz, ArH), 7.19 (d, 2H, *J* = 6.6 Hz, ArH), 7.45–7.69 (m, 3H, ArH), 7.72 (t, 3H, *J* = 8.4 Hz, ArH), 8.04 (d, 2H, *J* = 8.1 Hz, ArH), 8.15 (s, 1H, ArH), 8.76 (s, 1H, N=CH), 10.49 (s, 1H, CONH). Anal calcd for C_23_H_18_N_4_O_2_: C 72.24, H 4.74, N 14.65, O 8.37. Found C 72.27, H 4.70, N 14.70, O 8.41; MS *m*/*z* (M^+^) 382.

#### 2.9.6. N′-{(E)-[2-(4-Methylphenoxy)quinolin-3-yl]methylidene}phenoxymethyl-3-carbohydrazide **(34)**

Yield 71%. m.p. 188–190°C. IR (KBr) cm^−1^: 3245 (NH), 2879 (CH, aromatic), 1670 (C=O), 1463 (C=N), 1259 (C–O). ^1^H-NMR (300 MHz DMSO-d_6_) *δ* ppm: 2.35 (s, 3H, CH_3_), 5.24 (s, 2H, OCH_2_), 6.95 (d, 3H, *J* = 7.8 Hz, ArH), 7.14 (d, 2H, *J* = 7.5 Hz, ArH), 7.25 (d, 3H, *J* = 8.1 Hz, ArH), 7.48–7.65 (m, 4H, ArH), 8.00–8.09 (m, 1H, ArH), 8.49 (s, 1H, ArH), 8.87 (s, 1H, N=CH), 11.84 (s, 1H, CH_2_CONH). Anal calcd for C_25_H_21_N_3_O_2_: C 72.98, H 5.14, N 10.21, O 11.67. Found C 72.94, H 5.09, N 10.25, O 11.72; MS *m*/*z* (M^+^) 411.

#### 2.9.7. N′-{(E)-[2-(4-Methylphenoxy)quinolin-3-yl]methylidene}-3,5-dinitrophenyl-3-carbohydrazide **(35)**

Yield 75%. m.p. 70–72°C. IR (KBr) cm^−1^: 3278 (NH), 2887 (CH, aromatic), 1673 (C=O), 1465 (C=N), 1262 (C–O). ^1^H-NMR (300 MHz DMSO-d_6_) *δ* ppm: 2.37 (s, 3H, CH_3_), 6.93 (d, 2H, *J* = 7.8 Hz, ArH), 7.15 (d, 2H, *J* = 7.8 Hz, ArH), 7.25 (d, 2H, *J* = 8.4 Hz, ArH), 7.47–7.68 (m, 4H, ArH), 8.00–8.07 (m, 1H, ArH), 8.11 (s, 1H, ArH), 8.47 (s, 1H, N=CH), 12.14 (s, 1H, CONH). Anal calcd for C_24_H_17_N_5_O_5_: C 61.15, H 3.63, N 14.86, O 20.36. Found C 61.11, H 3.67, N 14.81, O 20.31; MS *m*/*z* (M^+^) 471.

#### 2.9.8. 2-Hydroxy-N′-{(E)-[2-(4-methylphenoxy)quinolin-3-yl]methylidene}benzohydrazide **(36)**

Yield 74%. m.p. 215-216°C. IR (KBr) cm^−1^: 3316 (OH), 3290 (NH), 2887 (CH, aromatic), 1672 (C=O), 1454 (C=N), 1265 (C–O). ^1^H-NMR (300 MHz DMSO-d_6_) *δ* ppm: 2.35 (s, 3H, CH_3_), 6.87–6.94 (m, 1H, ArH), 7.18–7.28 (m, 5H, ArH), 7.46–7.55 (m, 3H, ArH), 7.60 (d, 2H, *J* = 8.4 Hz, ArH), 7.76 (t, 2H, *J* = 11.4 Hz, ArH), 8.15 (s, 1H, N=CH), 8.84 (s, 1H, OH), 10.48 (s, 1H, CONH). Anal calcd for C_24_H_17_N_5_O_5_: C 61.15, H 3.63, N 14.86, O 20.36. Found C 61.11, H 3.67, N 14.81, O 20.31; MS *m*/*z* (M^+^) 397.

### 2.10. General Method for the Synthesis of 2-(4-Methylphenoxy)-3-(5-substituted-1,3,4-oxadiazol-2-yl)quinoline **(37–44)**

To an ethanolic solution of* substituted-(2-p-tolyloxy-quinolin-3-ylmethylene)-hydrazide* (0.01 mole)** (29-36)** and chloramin-T (0.01 mole) was added. The solution was refluxed for 4 h, and sodium chloride which separated out during the course of reaction was filtered off. Excess ethanol was completely removed from the filtrate by distillation under reduced pressure, leaving behind a solid mass which was crystallized from ethanol to give the desired compound. Recrystallize from ethyl alcohol.

#### 2.10.1. 3-[5-(Naphthalen-2-yloxymethyl)-[1,3,4]oxadiazol-2-yl]-2-p-tolyloxy-quinoline **(37)**

Yield 65%. m.p. 115-116°C. IR (KBr) cm^−1^: 2875 (CH, aromatic), 1498 (N=C), 1238 (C–O). ^1^H-NMR (300 MHz DMSO-d_6_) *δ* ppm: 2.40 (s, 3H, CH_3_), 5.52 (s, 2H, OCH_2_), 7.28–7.50 (m, 5H, ArH), 7.67 (d, 6H, *J* = 9.8 Hz, ArH), 7.82 (t, 3H, *J* = 20.7 Hz, ArH), 7.88 (d, 2H, *J* = 8.4 Hz). Anal calcd for C_29_H_21_N_3_O_3_: C 75.80, H 4.61, N 9.14, O 10.45. Found: C 75.85, H 4.57, N 9.19, O 10.38; MS *m*/*z* (M^+^) 459.

#### 2.10.2. 3-(5-Benzyl-1,3,4-oxadiazol-2-yl)-2-(4-methylphenoxy)quinoline **(38)**

Yield 54%. m.p. 147-148°C. IR (KBr) cm^−1^: 2972 (CH, aromatic), 1462 (N=C), 1237 (C–O). ^1^H-NMR (300 MHz DMSO-d_6_) *δ* ppm: 2.43 (s, 3H, ArH), 4.32 (s, 2H, CH_2_), 7.19 (d, 3H, *J* = 8.1 Hz, ArH), 7.26 (d, 3H, *J* = 7.2 Hz, ArH), 7.47 (t, 2H, *J* = 14.4 Hz, ArH), 7.68–7.77 (m, 3H, ArH), 7.89 (d, 2H, *J* = 6.8 Hz, ArH). Anal calcd for C_25_H_19_N_3_O_2_: C 76.32, H 4.87, N 10.68, O 8.13. Found: C 76.27, H 4.91, N 10.71, O 8.12; MS *m*/*z* (M^+^) 393.

#### 2.10.3. 2-(4-Methylphenoxy)-3-[5-(4-nitrophenyl)-1,3,4-oxadiazol-2-yl]quinoline **(39)**

Yield 57%. m.p. 85–88°C. IR (KBr) cm^−1^: 2867 (CH, aromatic), 1509 (N=C), 1341 (C–O). ^1^H-NMR (300 MHz DMSO-d_6_) *δ* ppm: 6.42 (t, 1H, *J* = 12.4 Hz, ArH), 7.02–7.35 (m, 5H, ArH), 7.47–7.67 (m, 3H, ArH), 7.72 (t, 1H, *J* = 14.4 Hz, ArH), 7.83 (d, 2H, *J* = 8.4 Hz, ArH). Anal calcd for C_24_H_16_N_4_O_4_: C 67.92, H 3.80, N 13.20, O 15.08. Found: C 67.97, H 3.78, N 13.23, O 15.04; MS *m*/*z* (M^+^) 424.

#### 2.10.4. 3-[5-(4-Chlorophenyl)-1,3,4-oxadiazol-2-yl]-2-(4-methylphenoxy)quinoline **(40)**

Yield 56%. m.p. 127–131°C. IR (KBr) cm^−1^: 2867 (CH, aromatic), 1509 (N=C), 1255 (C–O), 756 (C–Cl). ^1^H-NMR (300 MHz DMSO-d_6_) *δ* ppm: 2.34 (s, 3H, CH_3_) 7.18 (d, 2H, *J* = 8.1 Hz, ArH), 7.25 (d, 2H, *J* = 7.8 Hz, ArH), 7.52 (t, 2H, *J* = 9.3 Hz, ArH), 7.59 (d, 2H, *J* = 8.1 Hz, ArH), 7.73 (d, 2H, *J* = 7.2 Hz, ArH), 8.11 (d, 2H, *J* = 7.2 Hz, ArH), 8.47 (s, 1H, ArH). Anal calcd for C_24_H_16_ClN_3_O_2_: C 69.65, H 3.90, Cl 8.57, N 10.15, O 7.33. Found: C 69.62, H 3.93, Cl 8.56, N 10.11, O 7.27; MS *m*/*z* (M^+^) 413.

#### 2.10.5. 2-(4-Methylphenoxy)-3-[5-(pyridin-3-yl)-1,3,4-oxadiazol-2-yl]quinoline **(41)**

Yield 64%. m.p. 182–184°C. IR (KBr) cm^−1^: 2871 (CH, aromatic), 1444 (N=C), 1231 (C–O). ^1^H-NMR (300 MHz DMSO-d_6_) *δ* ppm: 3.27 (s, 3H, CH_3_), 7.12 (d, 2H, *J* = 8.4 Hz, ArH), 7.21 (d, 2H, *J* = 6.7 Hz, ArH), 7.44–7.64 (m, 3H, ArH), 7.75 (t, 3H, *J* = 11.4 Hz, ArH), 8.01 (d, 2H, *J* = 6.8 Hz, ArH), 8.07 (s, 1H, ArH). Anal calcd for C_23_H_16_N_4_O_2_: C 72.62, H 4.24, N 14.73, O 8.41. Found: C 72.56, H 4.27, N 14.68, O 8.46; MS *m*/*z* (M^+^) 380.

#### 2.10.6. 2-(4-Methylphenoxy)-3-[5-(phenoxymethyl)-1,3,4-oxadiazol-2-yl]quinoline **(42)**

Yield 77%. m.p. 99–102°C. IR (KBr) cm^−1^: 3358 (OH), 2865 (CH, aromatic), 1404 (N=C), 1305 (C–O). ^1^H-NMR (300 MHz DMSO-d_6_) *δ* ppm: 2.40 (s, 3H, CH_3_), 4.47 (s, 2H, CH_2_O), 7.14 (d, 2H, *J* = 11.4 Hz, ArH), 7.21–7.31 (m, 5H, ArH), 7.38 (d, 2H, *J* = 7.1 Hz, ArH), 7.47 (t, 2H, *J* = 14.4 Hz, ArH), 7.55 (d, 2H, *J* = 11.1 Hz, ArH), 7.79 (d, 2H, *J* = 8.1 Hz, ArH), 7.89 (s, 1H, ArH). Anal calcd for C_25_H_19_N_3_O_3_: C 73.34, H 4.68, N 10.26, O 11.72. Found: C 73.29, H 4.71, N 10.31, O 11.77; MS *m*/*z* (M^+^) 409.

#### 2.10.7. 3-[5-(3,5-Dinitrophenyl)-1,3,4-oxadiazol-2-yl]-2-(4-methylphenoxy)quinoline **(43)**

Yield 65%. m.p. 206–209°C. IR (KBr) cm^−1^: 2887 (CH, aromatic), 1465 (C=N), 1262 (C–O). ^1^H-NMR (300 MHz DMSO-d_6_) *δ* ppm: 2.33 (s, 3H, CH_3_), 6.97 (d, 2H, *J* = 8.1 Hz, ArH), 7.05 (d, 2H, *J* = 7.2 Hz, ArH), 7.22 (d, 2H, *J* = 9 Hz, ArH), 7.41–7.58 (m, 4H, ArH), 7.79–7.95 (m, 2H, ArH). Anal calcd for C_24_H_15_N_5_O_6_: C 61.41, H 3.22, N 14.92, O 20.45. Found C 61.37, H 3.22, N 14.91, O 20.44; MS *m*/*z* (M^+^); MS *m*/*z* (M^+^) 469.

#### 2.10.8. 2-{5-[2-(4-Methylphenoxy)quinolin-3-yl]-1,3,4-oxadiazol-2-yl}phenol **(44)**

Yield 74%. m.p. 145-146°C. IR (KBr) cm^−1^: 3178 (OH), 2922 (CH, aromatic), 1662 (C=O), 1440 (N=C), 1276 (C–O). ^1^H-NMR (300 MHz DMSO-d_6_) *δ* ppm: 2.34 (s, 3H, CH_3_), 7.07 (d, 2H, *J* = 8.1 Hz, ArH), 7.19–7.36 (m, 5H, ArH), 7.44 (d, 2H, *J* = 6.3 Hz, ArH), 7.52 (t, 2H, *J* = 17.4 Hz, ArH), 7.68 (d, 1H, *J* = 7.8 Hz, ArH), 7.75 (d, 1H, *J* = 7.2 Hz, ArH), 7.99 (d, 1H, *J* = 8.1 Hz, ArH). Anal calcd for C_24_H_17_N_3_O_3_: C 72.90, H 4.33, N 10.63, O 12.14. Found: C 72.95, H 4.37, N 10.64, O 12.17, MS *m*/*z* (M^+^) 395.

### 2.11. Anticancer Activity [35–40]

There were eleven compounds of the series, selected and screened for their anticancer activity for one-dose assay and among that two compounds were selected for 5-dose assay, after one-dose assay done by the National Cancer Institute (NCI) on leukemia, melanoma, lung, colon, CNS, ovarian, renal, prostate, and breast cancers cell lines, nearly 60 in number according to their screening protocol. All the synthesised compounds and structure of the compounds were submitted online to the official site of NCI for anticancer screening. Among 24 compounds only 11 compounds were selected for anticancer screening. NCI has its own selection procedure of the compounds for anticancer screening based on the novelty of heterocyclic ring system, drug-like properties utilizing the concept of privileged scaffolds, structure based on computer-aided drug design, and so forth, while the chemical structures containing some problematic linkages or functional groups. The anticancer screening was carried out as per the NCI US protocol. All compounds submitted to the NCI 60 Cell screen were tested initially at a single high dose (10^−5^ M) on leukemia, melanoma, lung, colon, CNS, ovarian, renal, prostate, and breast cancers cell lines, nearly 60 in number. The one-dose assay data was reported as a mean graph of the percent growth of treated cells. The number reported for the one-dose assay is the growth relative to the no-drug control and relative to the time zero number of cells. Using the absorbance measurements (time zero (Tz), control growth (C), and test growth in the presence of drug at the five concentration levels (Ti)) the percentage growth was calculated at each of the drug concentration levels.

Percentage growth inhibition is calculated as follows:

 [(Ti − Tz)/(C − Tz)] × 100 for concentrations for which Ti ≥ Tz.
 [(Ti − Tz)/Tz] × 100 for concentrations for which Ti < Tz.

Three dose response parameters are calculated for each experimental agent. The growth inhibition of 50% (GI50) is calculated from [(Ti − Tz)/(C − Tz)] × 100 = 50, which is the drug concentration resulting in a 50% reduction in the net protein increase (as measured by SRB staining) in control cells during the drug incubation. The drug concentration resulting in total growth inhibition (TGI) is calculated from Ti = Tz. The LC50 (concentration of drug resulting in a 50% reduction in the measured protein at the end of the drug treatment as compared to that at the beginning) indicating a net loss of cells following treatment is calculated from [(Ti − Tz)/Tz] × 100 = −50. Values are calculated for each of these three parameters if the level of activity is reached; however, if the effect is not reached or is exceeded, the value for that parameter is expressed as greater or less than the maximum or minimum concentration tested or if the effect exceeded the level of activity, the value of parameter was expressed as greater than the maximum concentration tested. Log GI50, log TGI, and log LC50 are the logarithm molar concentrations producing 50% growth inhibition (GI50), a total growth inhibition (TGI), and a 50% cellular death (LC50), respectively.

## 3. Result and Discussion

### 3.1. Chemistry

**Scheme 2:**
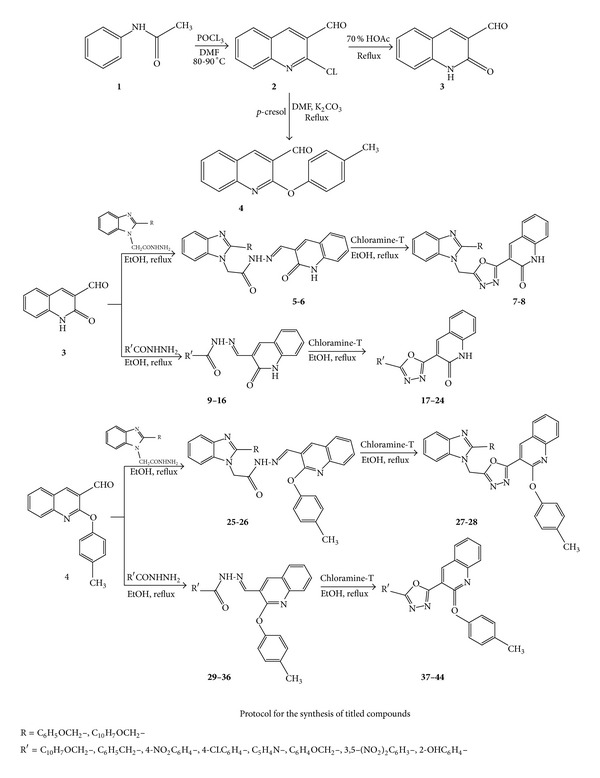


2-Chloroquinoline-3-carbaldehyde** 2** was prepared from acetanilide via Vilsmeier-Haack approach. 2-Oxo-1,2-dihydroquinoline-3-carbaldehyde** 2** has been prepared in the presence of 70% acetic acid, and 2-(p-tolyloxy)quioline-3-carbaldehyde** 4** was prepared from** 2** by using *p*-cresol and K_2_CO_3_ in the presence of DMF. In the step 1, 2-(2-phenoxy/naphthyloxy-1H-benzimidazol-1-yl)-N′-[(E)-(2-oxo-1,2-dihydroquinolin-3-yl)methylidene]acetohydrazide** 5**,** 6** have been prepared with the help of 2-[2-(phenoxy/naphthalen-2-yloxy methyl)-1H-benzimidazol-1-yl]acetohydrazide. The 3-[5-(2-Phenoxymethyl/naphthyloxy-benzoimidazol-1-ylmethyl)-[1,3,4]oxadiazol-2-yl]-1H-quinolin-2-one** 7**,** 8** were prepared by using 2-(2-phenoxy/naphthyloxy-1H-benzimidazol-1-yl)-N′-[(E)-(2-oxo-1,2-dihydroquinolin-3-yl)methylidene]acetohydrazide and chloramine-T. In the step 2 the* 2-(substituted)-(2-oxo-1,2-dihydroquinolin-3-yl)methylideneacetohydrazide* **9-16** *have been prepared by 1,2-dihydroquinoline and substituted aromatic hydrazide*. The corresponding 1,3,4-oxadiazole** 17–24** has been synthesised by chloramine-T and ethyl alcohol. 2-(2-Phenoxymethyl/naphthyloxymethyl-1H-benzimidazol-1-yl)-acetic acid (2-p-tolyloxy-quinolin-3-ylmethylene)-hydrazide** 25-26** have been synthesised from 2-[2-(phenoxy/naphthalen-2-yloxy methyl)-1H-benzimidazol-1-yl]acetohydrazide and 2-(p-tolyloxy)quioline-3-carbaldehyde** 4** and the corresponding 1,3,4-oxadiazole** 27-28** have also been synthesised by using chloramine-T. Further substituted-(2-p-tolyloxy-quinolin-3-ylmethylene)-hydrazide** 29–37** were synthesised by using aromatic hydrazide and the corresponding 1,3,4-oxadiazole** 38–44** from Schiff base have been synthesised. In general the 1H NMR spectra of the compound show one singlet between *δ* 5 to *δ* 6 CH_2_O. The Schiff bases explained the presence of –CONH (*δ* 10–12) and –N=CH from the presence of one singlet from *δ* 8 to *δ* 9. The next step of the synthesis of 1,3,4-oxadiazole, the disappearance of –CONH and –N=CH peaks confirm the formation of 1,3,4-oxadiazole (Scheme 2).

### 3.2. Anticancer Activity

Eleven compounds have been evaluated for their anticancer activity by NCI, USA. The observed anticancer screening of the compounds is given in Table 1. The two compounds were selected for five-dose assay; these compounds are given in Table 2. Anticancer data reveals that compound** 7** shows the growth percent ranges between 25.58 and 112.94, and the most sensitive cell lines are BT-549 (breast cancer) and T-47D (breast cancer) with the cell proliferation of 25.58 and 36.56, respectively. Compound** 17** shows the growth percent ranges from 66.38 to 119.29, and the most sensitive cell lines are UO-31 (renal cancer) and SNB-75 (CNS cancer) with the cell proliferation of 66.38 and 80.69, respectively. Compound** 18** shows the growth percent ranges from 7.52 to 120.39, and the most sensitive cell lines are HCT-116 (colon cancer), RPMI-8226 (leukemia), and CCRF-CEM (leukemia) with the cell proliferation of 7.52, 15.32, and 18.96, respectively. Compound** 20** shows the growth percent ranges from 58.38 to 168.13, and the most sensitive cell lines are UO-31 (renal cancer) and MCF7 (breast cancer) with the cell proliferation of 58.38 and 59.04, respectively. Compound** 21** shows the growth percent ranges from 73.22 to 322.59, and the most sensitive cell lines are SNB-75 (CNS cancer) and UO-31 (renal cancer) with the cell proliferation of 73.22 and 75.50, respectively. Compound** 22** shows the growth percent ranges from 68.36 to 116.40, and the most sensitive cell lines are UO-31 (renal cancer) and SNB-75 (CNS cancer) with the cell proliferation of 68.36 and 70.69, respectively. Compound** 24** shows the growth percent ranges from 71.08 to 132.21, and the most sensitive cell lines are UO-31 (renal cancer) and K-562 (leukemia) with the cell proliferation of 71.08 and 74.44, respectively. Compound** 27** shows the growth percent ranges from −18.17 to 94.56, and the most sensitive cell lines are HL-60TB (leukemia), MOLT-4 (leukemia), and SR (leukemia) with the cell proliferation of −18.17, −13.79, and −7.63, respectively. Compound** 38** shows the growth percent ranges from 22.17 to 107.52, and the most sensitive cell lines are CCRF-CEM (leukemia) and HCT-116 (colon cancer) with the cell proliferation of 22.17 and 22.22, respectively. Compound** 40** shows the growth percent ranges from 52.19 to 136.93, and the most sensitive cell lines are UO-31 (renal cancer) and HOP-92 (non-small cell lung cancer) with the cell proliferation of 52.19 and 65.04, respectively. Compound** 42** shows the growth percent ranges from 54.46 to 140.07, and the most sensitive cell lines are UO-31 (renal cancer) and SNB-75 (CNS cancer) with the cell proliferation of 54.46 and 65.12, respectively.

**Figure 1:**
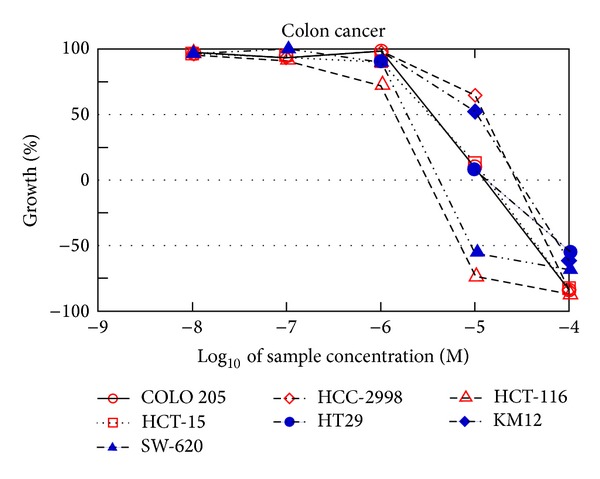


**Figure 2:**
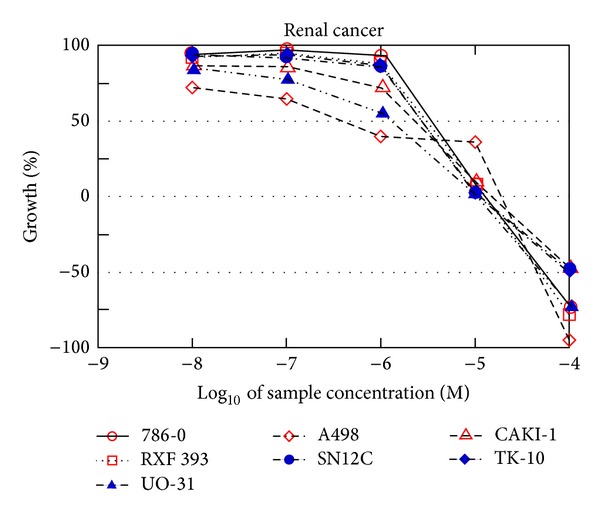


The result of anticancer activity of the five-dose assay of the selected compounds amongst their respected series is given in Table 2. Compound** 18** (NSC-776965) shows GI_50_ values ranges from 1.41 to 15.8 *μ*M, where the cell line of colon cancer recorded the best results with values ranging 10.4 *μ*M (Figure 1). Only two cancer cell lines presented TGI value with >100 *μ*M, and the value of the best result has been recorded on UO-31 (renal cancer) with value 3.72. In the 20 cell lines, Compound** 18** shows LC_50_ value with >100 03BCM. Compound** 27** (NSC-776971) shows GI_50_ values ranging from 0.40 to 14.9 *μ*M, where the cell line of renal cancer recorded the best results with values ranging between 0.40 and 3.91 *μ*M (Figure 2). Only one cancer cell line presented TGI value with >100 *μ*M, and the value of the best results has been recorded on HCT-116 (colon cancer) with the value of 1.64. In the 18 cell lines, Compound** 27** shows LC_50_ value with >100 *μ*M.

## 4. Conclusion

The novel series of 1,3,4-oxadiazole analogues have been synthesized in good yields and the anticancer activity showed good results in two compounds, that is,** 18**,** 27** (NSC 776965 and NSC 776971). The present studies reveal that compound** 27** is a potent lead compound for anticancer drug discovery and further research. The 1,3,4-oxadiazole anlogue provides a valuable new therapeutic intervention for the treatment of cancer disease.

## Figures and Tables

**Table 1:** Sixty human tumor cell lines anticancer screening data of 1,3,4-oxadiazole analogue.

Panel/cell line	Growth percent in one dose assay										
Compound (NSC code)	7 (776963)	17 (776964)	18 (776965)	20 (776966)	21 (776967)	22 (776968)	24 (776969)	27 (776971)	38 (776970)	40 (776973)	42 (776972)
Leukemia											
CCRF-CEM	62.29	97.80	18.96	96.39	97.48	95.05	96.04	0.73	22.17	88.72	91.95
HL-60(TB)	62.07	84.99	27.06	93.94	87.64	92.65	84.86	18.17	70.13	100.01	94.79
K-562	57.10	96.88	30.83	94.02	89.08	73.62	74.44	−3.18	24.16	86.70	92.41
MOLT-4	38.52	93.82	57.10	81.63	98.55	84.81	82.67	−13.79	58.84	80.98	80.79
RPMI-8226	46.61	103.90	15.32	90.25	105.14	99.66	91.19	−4.32	62.44	82.45	94.31
SR	43.32	100.48	31.61	79.12	108.64	92.79	86.39	−7.63	NT	75.27	85.97
Non-small cell lung cancer											
A549/ATC	68.28	102.26	83.29	99.50	101.27	100.60	103.01	81.28	82.82	88.52	100.80
HOP-62	75.35	94.46	82.64	93.91	99.00	91.48	94.50	49.80	74.55	92.66	87.05
HOP-92	48.46	101.70	76.22	93.69	99.25	96.45	101.84	14.22	29.71	65.04	73.35
NCI-H226	71.75	105.00	85.35	92.81	99.06	88.51	95.88	64.92	71.42	81.09	92.83
NCI-H23	75.93	93.51	74.68	92.88	98.68	87.99	94.37	66.54	72.86	86.03	87.52
NCI-H322M	93.43	106.90	92.29	85.97	107.44	NT	NT	92.32	78.67	88.95	107.35
NCI-H460	85.87	107.30	92.22	104.87	109.52	105.19	104.90	94.15	97.77	95.26	102.46
NCI-H522	63.25	100.15	71.20	83.30	89.60	102.67	95.45	70.22	75.33	90.31	96.41
Colon Cancer											
COLO 205	80.21	105.24	64.37	97.14	104.48	98.73	97.24	84.59	88.67	92.43	102.20
HCC-2998	85.93	99.22	78.89	105.11	101.20	98.41	99.35	64.49	91.02	99.13	102.31
HCT-116	52.32	98.66	7.52	86.71	107.15	88.92	93.41	−2.01	22.22	76.72	93.59
HCT-15	62.63	99.53	48.99	99.71	106.48	100.05	94.38	17.48	59.61	82.78	93.26
HT29	69.34	110.21	57.87	103.09	106.76	116.20	102.94	4.53	87.36	77.61	104.15
KM12	75.93	102.58	77.76	99.64	101.47	101.95	102.29	16.75	78.55	87.48	96.72
SW-620	88.64	110.90	44.97	108.56	111.64	104.62	105.48	18.07	35.64	94.63	105.16
CNS cancer											
SF-268	63.84	100.31	78.44	100.92	105.11	98.48	97.26	20.56	79.10	95.00	101.65
SF-295	76.15	101.14	99.71	100.44	101.59	104.07	103.01	92.01	99.41	97.56	99.73
SF-539	92.51	105.21	80.83	98.30	93.04	93.80	104.84	63.34	78.31	89.57	95.69
SNB-19	81.62	99.60	85.99	104.39	106.66	107.24	118.35	52.42	86.69	100.65	102.12
SNB-75	58.53	80.69	69.28	70.05	73.22	70.69	79.63	54.90	51.42	68.98	65.12
U251	82.46	103.79	72.36	98.35	97.23	91.91	98.31	42.97	71.06	88.68	97.88
Melanoma											
LOX IMVI	63.23	93.92	35.03	97.46	94.55	87.02	94.83	19.62	67.22	88.33	85.77
MALME-3M	77.64	91.22	55.80	95.90	93.74	99.30	92.28	72.49	98.95	123.55	99.75
M14	82.44	99.10	65.84	104.89	99.12	101.29	103.24	45.50	83.29	97.31	98.93
MDA-MB-435	70.53	104.12	52.86	100.61	104.79	104.95	102.28	50.75	90.57	100.57	101.98
SK-MEL-2	92.34	108.70	78.37	101.15	92.57	113.85	112.61	89.78	87.92	91.80	103.20
SK-MEL-28	112.94	112.37	101.46	115.05	107.29	116.40	116.82	90.94	107.52	110.01	104.89
SK-MEL-5	62.94	103.93	56.83	102.49	100.93	99.16	100.67	80.51	95.51	99.00	98.47
UACC-257	83.64	105.12	79.98	113.86	107.46	108.60	111.79	94.56	99.85	105.57	99.76
UACC-62	59.62	95.76	47.86	85.07	96.49	95.85	107.63	50.78	66.60	72.73	75.35
Ovarian cancer											
IGROV1	76.24	113.11	65.98	83.58	104.80	111.13	132.21	37.80	68.88	73.42	76.86
OVCAR-3	49.61	106.56	69.55	113.21	111.72	100.92	107.68	35.71	88.39	94.52	105.76
OVCAR-4	50.34	95.67	61.34	82.18	97.13	86.48	103.76	53.88	72.53	71.06	82.16
OVCAR-5	106.83	94.49	120.39	114.36	100.24	103.17	120.08	87.66	96.39	96.96	94.41
OVCAR-8	70.89	101.90	57.81	103.22	107.61	95.51	99.33	25.47	76.36	90.40	96.99
NCI/ADR-RES	62.13	101.99	89.85	93.91	103.13	90.74	95.32	34.60	68.12	85.72	94.68
SK-OV-3	90.18	3 97.22	92.78	91.03	97.60	88.65	103.94	77.34	81.98	87.32	89.99
Renal cancer											
786-0	40.52	98.54	58.27	93.10	99.88	93.03	100.61	62.73	70.97	90.18	93.77
A498	57.65	110.18	105.94	107.33	95.15	78.05	99.26	54.08	60.34	65.84	96.42
ACHN	78.17	106.04	65.89	94.78	109.02	105.23	103.76	49.86	74.96	88.72	92.40
RXF 393	75.91	111.91	76.07	101.43	109.85	103.21	105.90	60.28	87.66	91.37	98.87
SN12C	73.98	95.38	74.05	109.91	105.74	100.61	NT	29.44	86.85	91.91	91.68
TK-10	85.06	114.13	111.84	168.13	96.89	104.18	94.72	77.13	106.65	136.93	140.07
UO-31	34.06	66.38	39.98	58.38	75.50	68.36	71.08	8.45	23.47	52.19	54.46
Prostate cancer											
PC-3	58.32	96.94	65.48	82.75	98.08	90.66	86.35	46.23	71.58	76.86	83.03
DU-145	58.80	119.29	71.06	107.46	108.55	97.39	106.30	63.49	99.19	98.06	116.21
Breast cancer											
MCF7	50.02	93.06	20.47	59.04	91.23	91.22	88.77	11.59	39.72	84.08	85.66
MDA-MB-231/ATCC	54.03	96.65	79.63	91.54	120.58	104.47	105.94	47.68	61.51	78.25	71.62
HS 578T	79.72	110.45	90.07	104.25	132.59	89.18	100.48	72.43	86.82	93.19	97.10
BT-549	73.60	93.87	48.94	92.07	91.04	86.09	95.84	14.67	83.85	93.88	91.31
T-47D	25.58	86.17	42.22	78.22	95.06	86.86	93.64	48.89	55.68	74.73	82.45
MDA-MB-468	36.56	110.38	53.93	90.74	102.81	102.12	103.17	92.11	95.67	96.62	109.56

Mean	68.21	100.70	66.23	96.41	100.99	96.26	99.33	46.61	73.77	88.86	94.16
Delta	42.63	34.32	58.71	38.03	27.77	27.90	28.25	64.78	51.60	36.67	39.70
Range	87.36	52.91	112.87	109.75	59.37	48.04	61.13	112.73	85.35	84.74	85.61

**Table 2:** Five dose vitro testing results of compound.

Panel/cell line	18 (776965)			27 (776971)		
	GI_50_	TGI	LC_50_	GI_50_	TGI	LC_50_
Leukemia						
CCRF-CEM	1.70	7.79	>100	0.414	3.74	>100
HL-60(TB)	3.43	21.2	>100	2.85	7.88	>100
K-562	3.29	14.4	>100	2.59	>100	>100
MOLT-4	2.69	9.22	>100	2.18	7.89	>100
RPMI-8226	1.94	6.67	>100	2.39	8.45	>100
SR	2.03	10.9	>100	7.34	8.54	>100
Non-small cell lung Cancer						
A549/ATCC	15.7	>100	>100	8.49	24.2	62.3
HOP-62	12.6	34.3	93.5	2.98	13.0	52.2
HOP-92	3.34	15.3	53.3	1.27	5.37	29.2
NCI-H226	11.5	94.4	>100	5.99	31.4	>100
NCI-H23	11.3	39.5	>100	4.66	20.7	83.1
NCI-H322M	15.8	34.4	75.1	14.9	34.4	79.6
NCI-H460	6.63	28.3	>100	10.4	24.4	57.1
NCI-H522	2.27	5.90	26.7	6.34	21.1	55.6
Colon cancer						
COLO 205	3.55	13.2	44.9	1.89	4.44	12.3
HCC-2998	12.7	27.5	59.7	4.40	15.3	50.9
HCT-116	1.41	3.10	6.83	0.431	1.64	5.82
HCT-15	3.30	13.5	44.9	2.13	7.69	44.0
HT29	3.78	12.9	83.3	2.02	4.43	9.70
KM12	10.4	28.4	77.9	2.85	11.8	96.5
SW-620	1.87	4.16	9.25	1.08	4.79	32.4
CNS cancer						
SF-268	10.7	42.0	>100	3.41	15.9	69.8
SF-295	11.2	32.2	92.7	3.48	14.1	42.1
SNB-19	12.9	38.1	>100	3.78	17.0	63.4
SNB-75	6.07	21.4	53.5	2.16	9.74	45.8
U251	4.12	14.9	43.3	1.58	3.62	8.29
Melanoma						
LOX IMVI	1.74	4.30	13.1	1.59	4.32	17.1
MALME-3M	3.94	16.2	89.6	3.65	15.6	>100
M14	3.16	15.2	54.7	2.33	7.40	34.5
MDA-MB-435	3.51	17.5	77.8	3.06	11.9	40.5
SK-MEL-5	3.43	16.4	55.2	1.90	3.97	8.30
UACC-257	4.79	24.4	88.2	3.28	14.9	59.4
UACC-62	3.43	15.6	47.7	5.62	20.7	53.4
Ovarian cancer						
IGROV1	5.20	27.1	>100	3.25	22.0	>100
OVCAR-3	3.25	11.0	35.4	2.41	6.65	35.1
OVCAR-4	4.23	18.3	47.2	2.36	10.2	>100
OVCAR-8	2.71	NT	>100	2.12	7.52	>100
NCI/ADR-RES	14.4	71.1	>100	2.84	10.9	>100
SK-OV-3	18.3	38.1	79.3	11.8	32.7	90.3
Renal cancer						
786-0	4.01	14.6	58.2	3.09	11.8	51.4
A498	13.3	27.1	55.1	0.409	18.7	44.9
CAKI-1	3.65	14.4	48.2	2.29	14.9	>100
RXF 393	2.93	10.9	79.1	2.71	10.6	45.0
SN12C	3.46	28.2	>100	2.76	11.9	>100
TK-10	5.23	16.0	54.1	3.91	9.92	96.5
UO-31	1.72	3.72	8.05	1.19	10.2	48.4
Prostate cancer						
PC-3	4.16	70.6	>100	2.71	15.4	>100
DU-145	5.18	18.5	48.9	5.46	45.7	>100
Breast cancer						
MCF7	2.41	12.9	58.5	0.949	5.38	55.6
MDA-MB-231/ATCC	3.29	14.7	>100	1.64	4.62	70.7
HS 578T	13.3	>100	>100	4.39	33.8	>100
BT-549	2.56	10.2	34.2	1.77	4.06	9.31
T-47D	3.04	17.1	>100	2.61	14.6	>100
MDA-MB-468	2.29	9.87	59.2	3.95	18.3	74.4
